# Unique feeding profiles in children with pediatric feeding disorder and comorbid autism spectrum disorder: a retrospective cohort study

**DOI:** 10.1007/s00431-026-06920-8

**Published:** 2026-04-13

**Authors:** Tut Galai, Yael Goldberg, Alma Ziv, Nataly Kalamitzky, Kim Shemer, Nika Braiman, Shlomi Cohen, Hadar Moran-Lev

**Affiliations:** 1 Pediatric Gastroenterology Institute, “Dana-Dwek” Children’s Hospital, Tel Aviv Sourasky Medical Center, affiliated to the Gray Faculty of Medical and Health Sciences, Tel Aviv University, Tel Aviv, Israel; 2 Department of Pediatrics, “Dana-Dwek” Children’s Hospital, Tel Aviv Sourasky Medical Center, affiliated to the Gray Faculty of Medical and Health Sciences, Tel Aviv University, Tel Aviv, Israel

**Keywords:** Autism spectrum disorder, Pediatric feeding disorder, Infants, Toddlers

## Abstract

To compare the clinical characteristics, sociodemographic factors and feeding profiles of children aged 0–60 months diagnosed with pediatric feeding disorder (PFD) with and without comorbid autism spectrum disorder (ASD), in order to characterize features unique to each diagnosis. This retrospective comparative study included all children aged 0–60 months diagnosed with PFD between 2020 and 2023 at a tertiary pediatric center. Participants were categorized into 2 groups: those with comorbid ASD (ASD-PFD group) and those without ASD (PFD-only group), reflecting children diagnosed with ASD by age 5 years within the available follow-up. Clinical, demographic, perinatal and feeding-related data were extracted from medical records and analyzed. Among 141 participants, 47 were in the ASD-PFD group and 94 in the PFD-only group. The ASD-PFD group had a higher proportion of males (52.6% vs. 34.4%, *P* = 0.03) and cesarean deliveries (38% vs 23%, *P* = 0.03). These children were born to parents with lower educational attainment (*P* < 0.05) and presented (10 vs. 5 months) and were diagnosed (14 vs. 9 months) with PFD at older ages (P = 0.05). Nutritional dysfunction was more prevalent (55.6% vs 26.6%), whereas psychosocial dysfunction was less common (8.3% vs. 29.8%) in the ASD-PFD group (*P* = 0.007). Multivariable analysis identified male sex, cesarean delivery, and lower parental educational status as independent predictors of ASD, whereas psychosocial feeding dysfunction was inversely associated with ASD. *Conclusions*: Children with coexisting PFD and ASD exhibit a distinct profile characterized by later diagnosis and a predominance of nutritional over psychosocial feeding dysfunction, highlighting the importance of early recognition and tailored multidisciplinary care.

**Table:** 

What is Known: • *Pediatric feeding disorder (PFD) is common in children with autism spectrum disorder (ASD).* • *Most studies focus on older children; data on infants and toddlers are limited.*
What is New: • *Infants and toddlers with ASD and PFD show distinct profiles: later presentation and diagnosis, more nutritional and less psychosocial dysfunction.* • *In one-third, PFD preceded ASD diagnosis, highlighting feeding issues as early markers.* • *Lower parental education and higher cesarean rates were more common in ASD-PFD cases.*

What is Known:

• *Pediatric feeding disorder (PFD) is common in children with autism spectrum disorder (ASD).*

• *Most studies focus on older children; data on infants and toddlers are limited.*

What is New:

• *Infants and toddlers with ASD and PFD show distinct profiles: later presentation and diagnosis, more nutritional and less psychosocial dysfunction.*

• *In one-third, PFD preceded ASD diagnosis, highlighting feeding issues as early markers.*

• *Lower parental education and higher cesarean rates were more common in ASD-PFD cases.*

## Introduction

Pediatric feeding disorder (PFD) is a complex and heterogeneous condition that can impair a child’s nutritional intake, feeding skills, medical status, and psychosocial functioning [1]. It encompasses challenges, such as food selectivity, oral motor dysfunction, poor appetite, and disruptive mealtime behaviors with or without growth impairment. PFD has become an increasingly recognized diagnosis, with estimates suggesting that up to 20% of caregivers express concerns regarding their child’s feeding behaviors, leading to more frequent healthcare utilization [2–4].

Feeding difficulties are particularly prevalent among children with autism spectrum disorder (ASD), with reports of a prevalence as high as 70%, representing a rate significantly higher than that in non-ASD populations [5, 6]. These difficulties often manifest as rigid food preferences, refusal of specific food textures or colors, and sensitivity to sensory stimuli during mealtime [7, 8]. Population-based studies and national epidemiologic data report that the median age of ASD diagnosis is approximately 2 years, reflecting a trend toward earlier identification in recent years [9, 10]. These findings suggest that early childhood represents a critical window for identifying developmental and behavioral markers associated with ASD. Early identification of feeding challenges in this population is highly relevant, given that persistent feeding difficulties are associated with adverse developmental and nutritional outcomes [11, 12]. Therefore, characterizing PFD phenotypes within the critical developmental window of infancy and toddlerhood may help identify feeding characteristics associated with ASD. This may facilitate earlier diagnosis of PFD, earlier recognition ASD, enable more targeted therapeutic strategies and potentially improve long-term outcomes. Despite the high prevalence of feeding difficulties in ASD, most studies focus on children older than 2 years, leaving a gap in understanding the early manifestations of feeding difficulties that emerge in infants and toddlers who are later diagnosed with ASD [6, 13]. Prospective cohort studies have demonstrated that infants later diagnosed with ASD exhibit atypical feeding patterns during the first year of life, including breastfeeding difficulties, delayed acceptance of complementary foods, and early food selectivity [14–16]. Consistent with these findings, a recent systematic review reported higher rates of feeding aversion, restricted dietary variety, and sensory-related feeding challenges in infants and toddlers later diagnosed with ASD compared with neurotypical peers [6]. Nevertheless, these studies largely characterize feeding behaviors rather than applying the structured WHO-based framework of PFD and few directly compare infants and toddlers with PFD with and without comorbid ASD. Therefore, the aim of this study was to characterize the clinical, sociodemographic, and feeding-related differences between children diagnosed with PFD with and without coexisting ASD, according to the accepted WHO-based diagnosis.

## Methods

### Study design and patient population

This retrospective study included children aged 0 to 60 months who were referred to the multidisciplinary feeding clinic at the Institute of Pediatric Gastroenterology, Hepatology, and Nutrition, Dana-Dwek Children’s Hospital, Tel Aviv Medical Center, between January 2020 and January 2023. Eligible participants were diagnosed with PFD based upon the WHO consensus criteria [17]. Excluded were children with missing data, those whose final diagnosis did not meet PFD criteria, and those whose comorbid medical or neurodevelopmental conditions (excluding ASD) could independently account for feeding difficulties. The latter included genetic syndromes, cerebral palsy, gastrointestinal diseases, and other neurodevelopmental or psychiatric disorders (e.g., intellectual disability, ADHD, tic disorders).

The final study cohort was divided into 2 groups: children with comorbid ASD (ASD-PFD group) and those without ASD (PFD-only group). The study was approved by the institutional review board of the Tel Aviv Medical Center (Helsinki Committee, reference number TLV-0590–20). Informed consent was waived since all data were fully anonymized, and the study adhered to Good Clinical Practice (GCP) guidelines.

### Data collection

The Feeding Clinic team consisted of a pediatric gastroenterologist, a dietitian, speech and language pathologists, and a psychologist. All participants underwent a standardized multidisciplinary assessment during the initial visit, which included detailed history taking, anthropometric measurements, and evaluation of feeding behaviors. Individualized treatment plans were then developed and included dietary recommendations, referrals for therapeutic services (e.g., speech, occupational, and/or psychological therapy), and additional medical workup as needed.

Data were extracted from the hospital’s electronic medical records, which integrate clinical documentation and laboratory data from both hospital and health maintenance organization (HMO) records.

Sociodemographic characteristics included:

1. Sociodemographic characteristics
  Age at first medical presentation for feeding difficulties, age at formal PFD diagnosis, sex, family size, parental education and marital status, and socioeconomic status (SES).
2. Medical history—perinatal factors (e.g., gestational age, birth weight, delivery method, and complications), past hospitalizations, medication use in the year prior to referral and developmental milestones.
3. Feeding history—feeding method at birth, age of complementary food introduction and characteristics of feeding difficulties.

### Definitions and classification of study variables

Age at first medical presentation for feeding difficulties defined as the age at which the child was first evaluated in any medical setting (hospital or HMO clinic) due to feeding concerns documented in the medical record. Age at formal PFD diagnosis- defined as the age at which the multidisciplinary feeding clinic established the diagnosis of pediatric feeding disorder according to the WHO consensus criteria, The SES was determined by means of the Israeli Central Bureau of Statistics’ 2015 classification of residential areas, which scores clusters from 1 (lowest) to 10 (highest) based upon 14 indicators across demographics, education, income, and employment [18]. Parental academic background was classified by completion of a tertiary education. Low birth weight (LBW) was defined as < 2500 g. Pregnancy complications included any maternal or fetal risk factors (e.g., gestational diabetes, preeclampsia, intrauterine growth restriction, cholestasis, or multiple gestation). Delivery complications were defined as maternal fever, prolonged rupture of membranes, shoulder dystocia or perinatal asphyxia. Hospitalizations and medication use were assessed from birth until the time of the initial clinic visit.

ASD diagnoses were identified through the institutional electronic medical record system, which integrates documentation from both the tertiary hospital and the national HMOs, supplemented by parental reports at the time of data extraction. ASD was diagnosed following a multidisciplinary evaluation conducted in child development centers within HMOs or in tertiary pediatric hospitals. Diagnoses are established according to the *Diagnostic and Statistical Manual of Mental Disorders, Fifth Edition (DSM-5)* criteria by a child and adolescent psychiatrist, or a developmental pediatrician with formal training in child development. In addition, the diagnostic process typically includes assessment by a qualified psychologist (clinical, developmental, rehabilitation, or educational) [9, 19].

PFD was diagnosed for the study purpose by the clinic’s multidisciplinary team according to the WHO International Classification of Functioning, Disability and Health (ICF) framework [17]. The diagnosis required persistent feeding disturbance for at least 2 consecutive weeks that was inappropriate for age, not attributable to cultural practices or food insecurity and not attributable to an otherwise diagnosed eating disorder. Eligibility was confirmed through review of the multidisciplinary feeding assessment to ensure that the documented findings met the consensus definition of PFD. Diagnostic coding alone was not used for case identification.

PFD was subtyped by the clinical team as follows:

- Nutritional dysfunction: malnutrition, specific nutrient deficiencies, or reliance upon oral formula supplementation.
- Feeding skill dysfunction: Any need for texture modification of liquid or food (including oral or pharyngeal sensory-motor impairments that limit acceptance or tolerance of age-appropriate liquids or food textures, or sensitivity to characteristics such as flavor, temperature, viscosity or appearance), use of modified feeding position or equipment and/or use of modified feeding strategies.
- Medical dysfunction: anatomical conditions interfering with age-appropriate oral intake (e.g., cleft palate, absent swallowing reflex).
- Psychosocial dysfunction: Feeding difficulties arising from psychosocial factors within the feeding environment, child, caregiver or dyad, including active or passive disruptive mealtime behaviors, learned feeding aversions, stress/distress, food over selectivity, failure to advance to an age-appropriate diet despite adequate skills, grazing patterns, or maladaptive caregiver feeding practices.

Pediatric feeding disorder is typically multifactorial, reflecting the interaction between medical, nutritional, feeding skill, and psychosocial domains. Because impairment in one domain may contribute to dysfunction in another, many children present with overlapping features across domains. For the purposes of this study, in cases of overlapping PFD features, the predominant PFD subtype was assigned based upon team consensus. Disagreement was resolved by majority vote.

### Statistical analyses

Data were analyzed using SPSS version 27.0 (IBM Corp., Armonk, NY). All statistical tests were 2-sided, and a *P*-value < 0.05 was considered statistically significant. The Kolmogorov–Smirnov and Shapiro–Wilk tests were used to assess the normality of continuous variables. Normally distributed variables were presented as means ± standard deviations (SD), while non-normally distributed variables were summarized as medians with interquartile ranges [IQR]. Categorical variables were compared between the ASD-PFD and PFD-only groups by means of Pearson’s chi-square test or Fisher’s exact test, as appropriate. Continuous variables were analyzed using independent samples t-tests for normally distributed data or Mann–Whitney U tests for skewed data. To identify independent predictors of ASD among children diagnosed with PFD, a multivariable logistic regression analysis was conducted. ASD diagnosis (yes/no) served as the dependent variable. Independent variables were selected based on clinical relevance and significant associations in univariate analyses and included: child sex (male vs female), age at PFD diagnosis (months), delivery mode (cesarean vs vaginal), parental academic background and predominant PFD subtype. All variables were entered simultaneously. Results are presented as adjusted odds ratios (aOR) with 95% confidence intervals (CI). Model calibration was assessed using the Hosmer–Lemeshow goodness-of-fit test.

## Results

### Study population

**Fig. 1:**
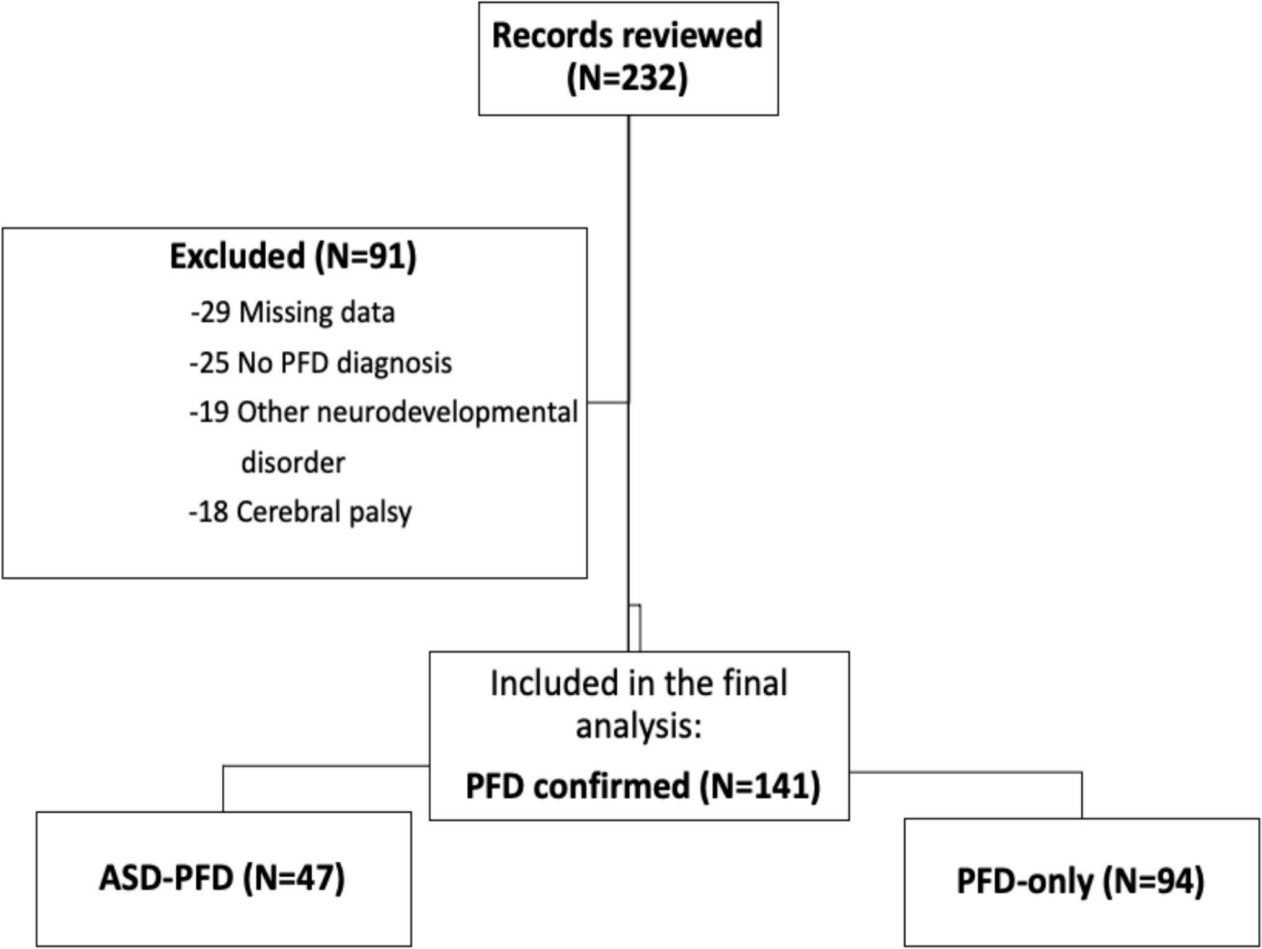
Flowchart of study population

A total of 232 medical records of children aged 0–60 months, diagnosed with PFD were reviewed. Ninety-one of them were excluded from the final analysis: 29 due to incomplete or missing data, 25 due to a revised diagnosis that excluded PFD, 19 due to comorbid neurodevelopmental syndromes and 18 due to a diagnosis of cerebral palsy. The final analytic cohort included 141 children with confirmed PFD (Fig. 1). Table 1 presents the demographic and clinical characteristics of the study participants with PFD stratified by ASD status. The median age of PFD presentation was 6 months [IQR: 4–15 months], 95 (67%) were males, and 47 (33.3%) had a comorbid diagnosis of ASD. Notably, the diagnosis of PFD preceded the diagnosis of ASD in 15 (32%) ASD-PFD participants.

**Table 1:** Demographic and clinical characteristics of children with pediatric feeding PFD with and without ASD

Variable	PFD (*n* = 141)	PFD with ASD (*n* = 47)	PFD -only (*n* = 94)	*P* value
Sex, male^a^	95 (67)	39 (83)	56 (59)	0.004**
Age at first medical presentation for feeding difficulties, months^b^	6 (4,15)	10 (5,20.25)	5 (3.62,10.25)	0.05*
Age at formal PFD diagnosis	11 (3,14)	14 (2,20)	9 (4,15)	0.05*
Socioeconomic status—cluster	7.5 (5,8)	6.5 (5,8)	8 (5,8)	0.23
Socioeconomic status—index	1.02 (0.38,1.08)	0.68 (0.3,1.08)	1.08 (0.38,1.08)	0.15
Parent marital status				
Married	113 (80)	37 (78)	76 (80)	0.54
Divorced	20 (14)	6 (12)	14 (15)	
Single parent	8 (5.6)	4 (8.5)	4 (4.2)	
Academic background 1 st parent	71 (50)	14 (29)	57 (61)	***0.002***
Academic background				
2nd parent	75 (53)	12 (25)	63 (67)	< 0.001***
Siblings, n	1 (0,2)	1 (0,2)	1 (0,1.5)	0.19
Gestational age, weeks	38 (37,39)	38 (35.2,39)	38 (37,39)	0.35
Delivery by C/S	41 (29)	19 (40)	22 (23)	***0.03***
Birth weight, grams	3011.5 (2643.75,3420)	3000 (2155,3446.5)	3042.5 (2705,3405)	0.43
Breastfeeding	51 (36)	21 (44)	30 (32)	0.06
Breastfeeding duration, months	5 (0,9)	3 (0,9.75)	5 (0,9)	0.74
Age at complementary food intro., months	6 (5,7)	6 (5,8)	6 (5,7)	0.96
Hospitalization	46 (32)	15 (32)	31 (33)	0.52
Gastrostomy tube feeding	7 (5)	4 (8)	3 (3)	0.168

a Values are expressed as n (%) for categorical variables

b Median and interquartile range (IQR) for continuous variables

^*^P < 0.05

^**^P < 0.01

^***^P < 0.001

*SD* standard deviation, *C/S* cesarean section, *PFD* pediatric feeding disorder, *ASD* autistic spectrum disorder

The children in the ASD-PFD group were significantly more likely to be males (83% vs. 59%, *P* = 0.004) and were more often born via cesarean Sect. (40% vs 23% for the PFD group, *P* = 0.03). Moreover, those with ASD-PFD were more often born to parents with lower educational attainment compared to the PFD group (71% vs 39% for 1 st parent, P = 0.002, and 75% vs 33% for the second parents, P < 0.001). Other sociodemographic characteristics, including SES and family composition, did not differ significantly between groups.

**Fig. 2:**
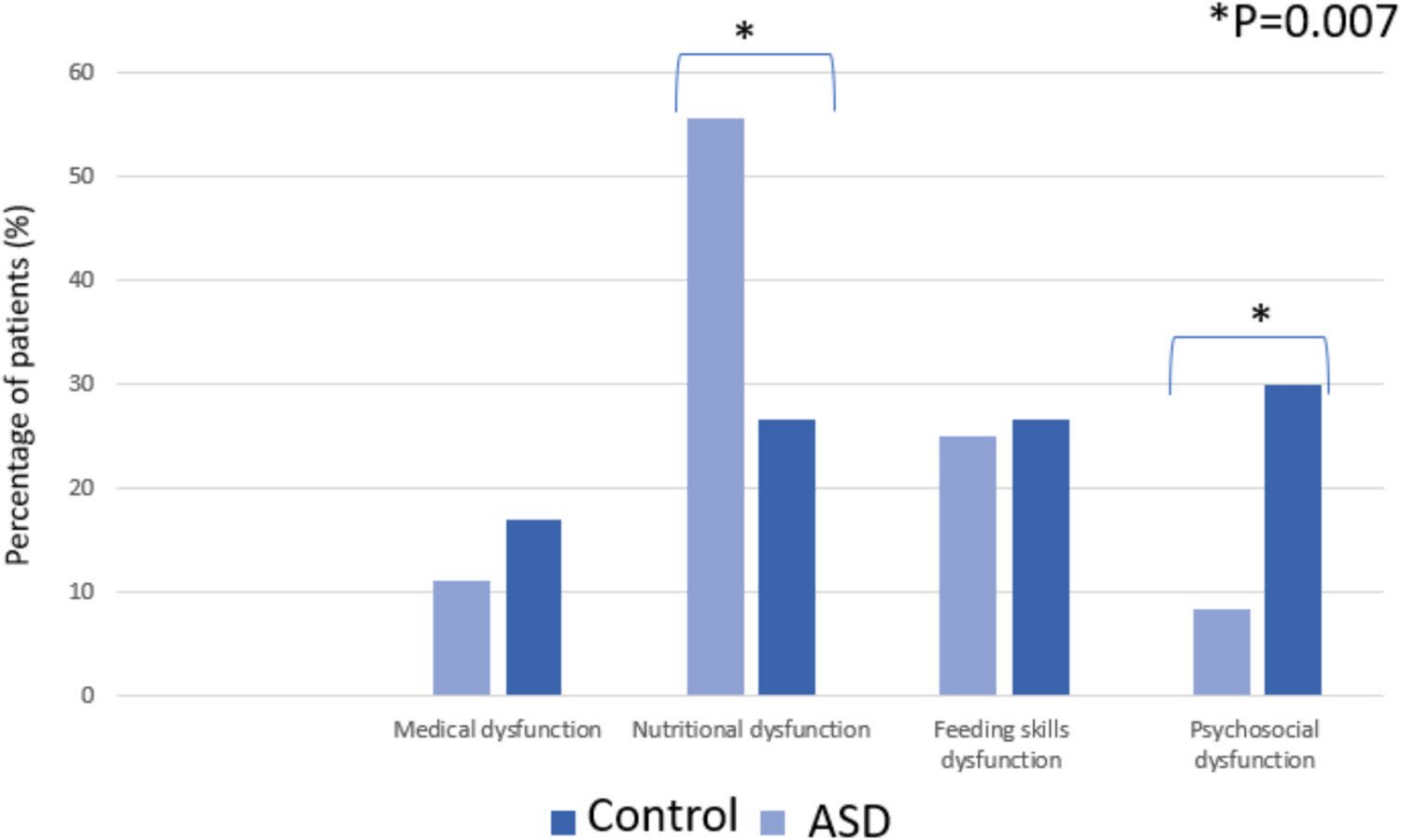
Comparison of feeding disorder subtypes by autism spectrum disorder status

Children with ASD presented for medical evaluation of feeding difficulties at an older age compared with their neurotypical peers (median 10 vs. 5 months, respectively, P = 0.05). The median age at formal PFD diagnosis in the multidisciplinary feeding clinic was 14 months in the ASD-PFD group and 9 months in the PFD-only group, P = 0.05), reflecting the interval between initial clinical presentation and multidisciplinary evaluation. There were no significant group differences in birth weight, gestational age, medication use, hospitalizations, or rates of gastrostomy tube placement. There was a trend toward higher breastfeeding rates in the ASD group that did not reach a level of significance. A comparison of PFD subtypes revealed that the infants and toddlers with ASD demonstrated significantly higher rates of nutritional dysfunction and lower rates of psychosocial dysfunction compared to those without ASD (*P* = 0.007; Fig. 2). In the multivariable logistic regression model, cesarean delivery, male sex, lower parental educational background, were associated with substantially increased odds of ASD. Regarding feeding disorder subtypes, psychosocial dysfunction was strongly and inversely associated with ASD. Although nutritional dysfunction was more frequent among children with ASD in the unadjusted analysis, its association with ASD was attenuated and no longer statistically significant after adjustment for other covariates in the multivariable model (Table 2).

**Table 2:** Multivariable Logistic Regression: Predictors of ASD Among Children with PFD

Variable	Adjusted OR	95% CI	*p*-value
Male sex	0.14	0.03–0.61	0.008**
Cesarean delivery	4.38	1.28–14.97	0.018*
Age of feeding disorder started- months	1.04	0.995–1.087	0.085
High parental educational background	0.25	0.08–0.79	***0.018***
Predominant nutritional dysfunction	0.23	0.04–1.27	0.093
Predominant psychosocial dysfunction	0.03	0.002–0.27	***0.002***

^*^P < 0.05

^**^P < 0.01

^***^P < 0.001

*ASD* autistic spectrum disorder, *PFD* pediatric feeding disorder

**Reference categories:** female sex, vaginal delivery, lower parental educational background, and absence of the specified predominant PFD subtype. Parental educational background was entered into the model as a composite variable and was classified as **high** when one or both parents had a high educational background. Adjusted odds ratios therefore represent the association of each variable relative to its reference category after multivariable adjustment

## Discussion

In the current study, based on a large database of children diagnosed with PFD, we identified several characteristics that distinguish children with ASD. Children with PFD and ASD were more likely to be male, to have been delivered by cesarean section, presented and diagnosed with PFD at an older age. Regarding the type of feeding disorder, children with ASD are presented more frequently with nutritional dysfunction and markedly less often with psychosocial dysfunction.

The finding that children with ASD and PFD presented for medical evaluation of feeding difficulties at an older age than children with PFD alone and were subsequently diagnosed with PFD later, requires careful interpretation. PFD may emerge at different developmental stages as children transition to complementary feeding and encounter increasing dietary complexity [20]. However, accumulating evidence suggests that feeding disturbances in children later diagnosed with ASD often begin early in life. Prospective longitudinal studies demonstrate that infants subsequently diagnosed with ASD exhibit feeding differences during the first year of life, including breastfeeding difficulties, delayed acceptance of complementary foods, limited dietary variety, and reluctance to try new foods, with these difficulties becoming more pronounced during the transition to solid foods and continuing to increase between 15 and 36 months of age [6, 21]. These findings suggest that feeding difficulties in children with ASD may frequently be present early but become more clinically apparent as developmental expectations for feeding expand. In this context, the later age of clinical presentation and diagnosis observed in our ASD cohort may partly reflect delayed recognition rather than a genuinely later onset of feeding pathology. Several mechanisms may contribute to this delay. Feeding difficulties in children with ASD often overlap with core neurodevelopmental features such as sensory sensitivities, communication difficulties, and behavioral rigidity. As a result, feeding problems may be attributed to autism itself rather than recognized as a distinct clinical condition. [6, 9, 22, 23]. This diagnostic overshadowing, combined with the broader diagnostic trajectory of ASD, where families often prioritize developmental evaluation and intervention, may delay referral for specialized feeding assessment. In addition, parents managing multiple behavioral and developmental challenges may initially perceive feeding difficulties as an expected component of autism rather than a separate medical concern.

Early identification of feeding difficulties is crucial given that persistent feeding problems are strongly associated with poor adaptive functioning and developmental delays [24]. Delayed recognition of PFD in children with ASD has been linked to increased nutritional inadequacy, greater food selectivity, and prolonged feeding difficulties extending into later childhood and adolescence, with significant implications upon both physical and mental health [13, 25, 26]. Interestingly in our study, PFD was diagnosed before ASD in one-third of our ASD-PFD group, emphasizing the importance of heightened developmental surveillance when persistent feeding issues are identified in infancy [6, 13, 27].

An additional finding in our study was the higher frequency of cesarean delivery among children in the ASD-PFD group. This observation is consistent with studies that reported a possible association between cesarean birth and increased risk for ASD [28, 29]. Hypothetical mechanisms responsible for that association include alterations in neonatal microbiome colonization, reduced hormonal exposure during labor, and delayed breastfeeding initiation, each of which may affect both feeding behavior and neurodevelopment [30–33]. While causality remains unconfirmed, this association highlights the importance of close feeding and developmental monitoring among cesarean-born infants, particularly those with additional risk factors.

We also observed important differences in PFD subtype distribution. Children with ASD exhibited significantly more nutritional dysfunctions and fewer psychosocial feeding challenges compared to their peers without ASD. In a multivariate analysis psychosocial dysfunction was strongly and inversely associated with ASD, reflecting the rarity of psychosocial feeding patterns among children with ASD. These findings reflect the predominance of sensory-related food selectivity and rigid eating patterns in ASD, which are typically driven by atypical sensory processing rather than psychosocial or environmental factors [34]. Previous studies have documented high rates of food selectivity and sensory-based food preferences in children with ASD, which may lead to nutritional deficiencies [35, 36]. In contrast, feeding difficulties among children without ASD are more often linked to maladaptive family dynamics, parental anxiety, or environmental stressors [1]. These differences have important implications for treatment. Traditional feeding interventions often target caregiver-child interactions and psychosocial contributors [37]. However, treatment approaches for children with ASD must be tailored to address the underlying sensory and behavioral drivers of feeding dysfunction. Considerable evidence supports the use of sensory integration strategies and structured behavioral therapies in this population. A multidisciplinary model that incorporates pediatric gastroenterology, psychology, and occupational and speech therapy has been shown to improve feeding outcomes in ASD, particularly when individualized for each child’s sensory and behavioral profile [38–41].

Our findings also reveal notable sociodemographic disparities. The parents of children in the ASD-PFD group were more likely to have lower educational achievement. This aligns with earlier reports demonstrating that lower parental education is associated with delayed recognition and underdiagnosis of developmental disorders, including ASD and feeding disorders [42, 43]. Health literacy is a key component of education level that affects caregivers’ ability to recognize developmental concerns, navigate complex healthcare systems, and access early intervention services [44, 45]. Compounding this, families with lower education levels may face additional barriers, such as transportation challenges, job inflexibility, or limited access to specialized care [46]. These findings underscore the need for targeted public health strategies, including universal developmental screening, integrated feeding assessments in routine pediatric care, and culturally tailored education for families in underserved communities.

To the best of our knowledge, this is one of the first studies to characterize the association between ASD and PFD in the first years of life, using the recent WHO-based consensus definition of PFD. Our results support the view that PFD in children with ASD represents a distinct clinical phenotype requiring specialized management. Clinically, these findings emphasize the value of early ASD screening among infants and toddlers presenting with nutritional dysfunction and minimal psychosocial challenges. Individualized interventions that address neurodevelopmental characteristics are likely to improve both feeding and developmental outcomes. This study has several limitations. Its retrospective design precludes establishing causal relationships between ASD and PFD phenotypes and therefore allows only the identification of associations between these conditions. Second, because the study relied on retrospective clinical records, the exact onset of PFD symptoms could not be determined; therefore, the age variables reported reflect the timing of first medical presentation and multidisciplinary diagnosis rather than the true onset of feeding difficulties. Some discordance may exist between clinical impressions and formal criteria for PFD subtypes, although diagnoses were made by an experienced multidisciplinary team using standardized criteria. Third, ASD classification was based on diagnoses documented in the institutional electronic medical record at the time of data extraction. Accordingly, our findings primarily reflect patterns among children in whom ASD had been recognized by age 5 years within the available follow-up period. As a result, a small degree of diagnostic misclassification is possible, and some co-occurring neurodevelopmental conditions, particularly ADHD and mild intellectual disability, may not yet have been fully identified or documented.

In conclusion, this study adds to the limited but growing body of evidence on feeding difficulties in children with ASD. The distinct clinical profile observed in this population, including later presentation and diagnosis, greater nutritional dysfunction, and fewer psychosocial contributors, highlights the need for early, tailored screening and intervention strategies. Given the potential long-term developmental and nutritional consequences of untreated feeding disorders, early recognition and individualized neurodevelopmentally informed care are essential for children at risk. Longitudinal prospective studies are needed to further validate the observed associations and to explore causality as well as to better assess the outcome of specific therapeutic modalities in this population.

## Data Availability

The datasets used and/or analyzed during the current study are available from the corresponding author on reasonable request.

## Abbreviations

ADHD: Attention-deficit/hyperactivity disorder
aOR: Adjusted odds ratio
ASD: Autistic Spectrum Disorder
ASD-PFD: Pediatric feeding disorder with comorbid autism spectrum disorder
CI: Confidence interval
CS: Cesarian Section
DSM-5: Diagnostic and Statistical Manual of Mental Disorders, Fifth Edition
GCP: Good Clinical Practice
HMO: Health maintenance organization
ICF: International Classification of Functioning, Disability and Health
IQR: Interquartile range
LBW: Low birth weight
PFD: Pediatric feeding disorder
SD: Standard deviation
SES: Socioeconomic status
WHO: World Health Organization
